# Lung Abscess and Recurrent Empyema After Infection With *Mycoplasma hominis*: A Case Report and Review of the Literature

**DOI:** 10.1093/ofid/ofab406

**Published:** 2021-08-04

**Authors:** Isabelle Moneke, Daniel Hornuss, Annerose Serr, Winfried V Kern, Bernward Passlick, Oemer Senbaklavaci

**Affiliations:** 1 Department of Thoracic Surgery, Medical Center – University of Freiburg, Freiburg, Germany; 2 Faculty of Medicine, University of Freiburg, Freiburg, Germany; 3 Division Cancer Research, Department of Thoracic Surgery, Medical Center – University of Freiburg, Freiburg, Germany; 4 Institute of Infectious Diseases, Department of Medicine, Medical Centre-University Hospital of Freiburg, Freiburg, Germany; 5 Institute of Microbiology and Hygiene, Medical Center – University of Freiburg, Freiburg, Germany; 6 German Cancer Consortium (DKTK), Partner Site Freiburg, German Cancer Research Center (DKFZ), Heidelberg, Germany

**Keywords:** empyema, immunocompromised patients, lung abscess, *Mycoplasma hominis*, sepsis

## Abstract

*Mycoplasma hominis* is a rarely identified cause of respiratory infection that can cause significant morbidity and mortality in immunocompromised patients. It is often missed due to the difficult detection of the organism with routine laboratory methods.

We present the case of a 63-year-old male with a history of lymphoma who was transferred to our hospital with recurrent right-sided empyema and lung abscess in the right lower lobe. Advanced microbiological analysis finally revealed infection with *M hominis*. Despite appropriate antibiotic treatment, prolonged drainage as well as repeated surgery, which eventually resulted in right lower bilobectomy, were necessary for clinical improvement of our patient.

Infection with *M hominis* may be more prevalent than previously indicated and can cause severe morbidity and mortality in thoracic surgery patients. Due to the diagnostic challenge, the appropriate antimicrobial treatment is often delayed. Inherent resistance to macrolides and inactivity of cell wall-active agents potentially complicate empiric antibiotic therapy. A review of the currently available literature enables a better understanding of the diagnostic difficulties and importance of this infection.

*Mycoplasma hominis* is a small bacterium that usually colonizes the human genitourinary tract by adhering to mucosal epithelial cells [1]. It can also be found in the respiratory secretions of 1% to 3% of healthy adults and approximately 8% of patients with chronic respiratory disease [2], eg, chronic obstructive pulmonary disease (COPD), asthma, or chronic bronchitis [3, 4]. Its role as a pathogen is usually limited to the structures of the urogenital system [5]. Although disseminated disease is rare, there are several reports of multiple sites of infection outside the genitourinary tract [6–8] such as “culture-negative” wound infections, mycoplasma endocarditis [9], or mediastinitis in cardiothoracic surgery patients [10]. Over the last decade, infection with *M hominis* has been well recognized in immunocompromised patients, such as lung or other solid organ transplants or patients with acquired immunodeficiency syndrome. It has also been detected in critically ill patients in the intensive care unit [11] or patients with hematologic malignancies [12-14]. Very few cases have been described in immunocompetent patients [15].

The identification in clinical specimens is often hindered by the fact that routine microbiological methods, for example, Gram staining, fail to detect this group of pathogens because they lack a cell wall [13, 16, 17]. Furthermore, the presence of *M hominis* in cultures can easily be missed because it grows very slowly, needs a cholesterol-rich medium, and the colonies are translucent [13]. Improved detection methods such as polymerase chain reaction (PCR) or immunohistochemistry are widely available but not routinely used in the conventional analysis of clinical specimens.

Most of the typical broad-spectrum antimicrobial agents are ineffective against *M hominis* [18]. Mycoplasmas are inherently resistant to beta-lactam-based (eg, penicillins, cephalosporins and carbapenems) or glycopeptide antibiotics (eg, vancomycin) as well as antimetabolites (eg, trimethoprim/sulfamethoxazole) or lipopolysaccharide-targeting antibiotics (eg, polymyxin) [18, 19] because they lack a cell wall. Moreover, *M hominis* is unique in its resistance to macrolides (eg, azithromycin), because the sequence of the *M hominis* single copy of 23S ribosomal ribonucleic acid has a G2057A transition, leading to a replacement of a guanidine with an adenine at a position known to abrogate macrolide binding to the ribosome [20].

The delay in diagnosis and appropriate treatment may lead to prolonged hospitalization, which may eventually lead to a significant increase in morbidity and mortality, especially in immunosuppressed or predisposed patients.

## CASE REPORT

**Figure 1.**
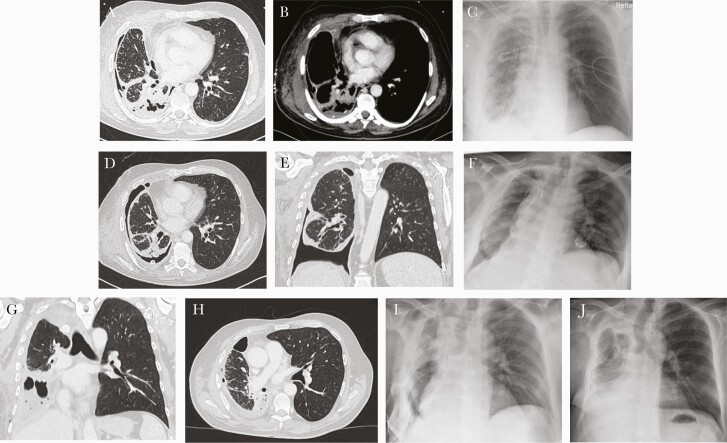
Initial computed tomography scan (a and b) and postoperative x-ray (c) after rethoracotomy and pleurectomy/decortications. Computed tomography scan 4 weeks later with persistent bronchopleural fistula and re-empyma (d and e) + postoperative x-ray after right lower lobectomy (f). Computed tomography scan another 4 weeks later with re-empyema and bronchial stump insufficiency (g and h) and postoperative x-ray after middle lobe lobectomy + latissimus dorsi flap (i). Final x-ray after discharge 3 weeks later (j).

A 63-year-old male with a history of fever and cough as well as chest pain for a week presented to a local hospital and was diagnosed with pneumonia in the right lower lobe. Over the next 2 weeks, he developed right-sided pleural fluid and recurrent fever up to 40°C. The laboratory tests showed an elevated leucocyte count as well as elevated blood levels of C-reactive protein. Eventually, right-sided empyema was diagnosed. Antibiotic therapy, first with piperacillin/tazobactam and then with meropenem, was administered for a total of 14 days. Extensive microbiological sampling was carried out. Multiple sets of blood cultures, pleural effusion, sputum, and urine samples were analyzed; however, no pathogen was identified. After treatment with drainage alone was not successful anterolateral thoracotomy was performed to evacuate the empyema. Again, no pathogen was detected in the tissue samples in the external laboratory. He had a history of stage IV follicular lymphoma and had been on maintenance therapy with rituximab until March 2020. Histology samples revealed no recurrence of lymphoma. After initial improvement after surgery, the patient deteriorated again and developed high fevers up to 40°C accompanied by hemodynamic instability and respiratory failure, despite continued antibiotic therapy. A computed tomography (CT) scan revealed recurrent empyema and lung abscess in the right lower lobe (Figure 1). He was referred to our hospital for further treatment. Repeat surgery was performed to evacuate the empyema the day after, which was approximately 3 weeks after initial presentation at the peripheral hospital. *Mycoplasma hominis* was isolated from pleural effusion and in the blind subcultures at the end of 6 days’ incubation of the blood cultures. Massive amounts of *M hominis* grew from the intraoperatively taken lung tissue samples. Subsequent PCR analysis of the tissue samples obtained during the first surgery retrospectively came up positive for *M hominis* as well (Table 1). Due to intrinsic resistance towards macrolides, antibiotic therapy with levofloxacin was initiated. After approximately 3 weeks he developed a fever again. Due to persisting detection of *M hominis* in cultures from pleural fluids and wound infection, at least partial resistance to fluoroquinolones was suspected, and combination therapy with doxycycline was administered. Susceptibility testing of the prevalent *M hominis* strain was performed in the reference laboratory for *Mycoplasma* and *Chlamydia* infections, showing no resistance towards fluoroquinolones and tetracyclines. Because infection with *M hominis* as well as *Ureaplasma* species is associated with hyperammonemia, the patient’s serum ammonia level was tested. It was found to be within the normal range 25 μmol/L (reference range, 20–65 μmol/L). There were no neurological symptoms.

**Table 1.** Detection of *M hominis* in Various Samples and Tissues Over the Time. Cobl = Columbia blood agar

Date	Sample	Pathogen	Bacterial Count	Growth in days	Comment	PCR result	Copies/ml
22 July 2020	Tissue lung (paraffin)					M. hominis	46,000
22 July 2020	Tissue lung (paraffin)					M. hominis	< 10
29 July 2020	Pleural fluid	M. hominis	plenty	2	on Cobl		
30 July 2020	Blood culture	M. hominis			blind subculture day 6		
30 July 2020	Blood culture	M. hominis			blind subculture day 6		
30 July 2020	Blood culture	M. hominis			blind subculture day 6		
30 July 2020	BAL	M. hominis	massive growth	4	on Cobl		
		K. pneumoniae	isolated				
30 July 2020	Tissue lung	M. hominis	massive growth	2	on Cobl	M. hominis	1.36 Mio
30 July 2020	Tissue lung	M. hominis	massive growth	2	on Cobl	M. hominis	600,000
30 July 2020	Tissue lung	M. hominis	massive growth	2	on Cobl	M. hominis	300,000
30 July 2020	Tissue lung	M. hominis	plenty	4	on Cobl	M. hominis	33,000
04 August 2020	Tissue parietal pleura	M. hominis	plenty	3	on Cobl	M. hominis	7,000
		S. epidermidis	enrichment				
04 August 2020	Tissue parietal pleura	M. hominis	plenty	3	on Cobl	M. hominis	69,000
		S. epidermidis	sporadic				
12 August 2020	Pleural fluid	M. hominis	plenty				
		E. faecium	sporadic				
		S. epidermidis	sporadic	3	on Cobl		
25 August 2020	Swab chest tube	E. faecium	enrichment				
		Hautflora	sporadic				
27 August 2020	Swab chest tube	M. hominis	sporadic	2	on Cobl		
27 August 2020	Tissue lung	M. hominis	sporadic	8	on Cobl		
08 September 2020	Tissue parietal pleura	no growth					
08 September 2020	Tissue parietal pleura	S. epidermidis	enrichment				
		C. acnes	sporadic				
25 September 2020	Pleural fluid	S. mitis/oralis	massive growth			M. hominis	< 10 pro ml
15 October 2020	DNA, parietal pleura (paraffin)						weak positive

Despite appropriate antibiotic treatment, repeated surgery to evacuate the empyema was necessary as well as vacuum-assisted closure of the wound. Due to persistent bronchopleural fistula, the patient finally underwent right lower lobectomy 3 weeks later. He significantly improved clinically and was discharged approximately 6 weeks after his admission at our hospital. Antibiotic therapy with levofloxacin and doxycycline was continued orally.

**Figure 2.**
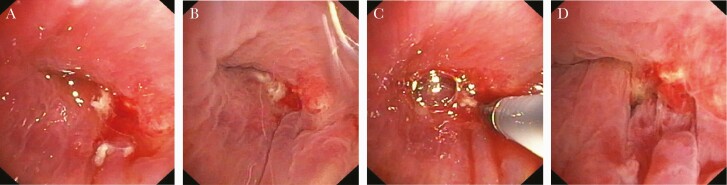
Bronchoscopy images showing bronchial stump insufficiency 4 weeks after right lower lobectomy.

It is unfortunate that at the first follow-up approximately 1 week later, he presented with elevated blood infection parameters and fever. The CT scan revealed pleural effusion and entrapped air, highly suggestive of re-empyema and bronchus stump insufficiency, which was confirmed in bronchoscopy (Figure 2). *Mycoplasma hominis* deoxyribonucleic acid could still be detected in the pleural fluid, despite ongoing antibiotic treatment, although in a much lower quantity of <10 copies per milliliter pleural fluid. Middle lobe lobectomy was necessary, and the bronchus stump was augmented with a musculus latissimus dorsi flap. After the final chest drain was removed, the patient was discharged to a rehabilitation facility.

**Figure 3.**
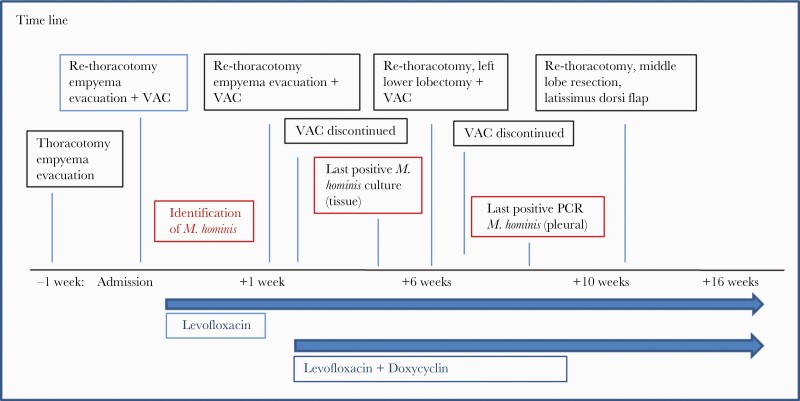
Time line of the surgeries required as well as antimicrobial therapy until recovery. *M. hominis*, *Mycoplasma hominis*; PCR, polymerase chain reaction; VAC, vacuum-assisted closure.

The patient was gradually recovering and regaining his strength over time. The last follow-up took place on November 2, 2020. Since there was no need for further treatment of the lymphoma at this time, because there was no indication of recurrence, antibiotic treatment was continued orally until November 26, 2020, 3 months after the last detection of viable *M hominis* (Figure 3).

### Patient Consent Statement

Consent for publication of individual data has been obtained from the patient.

## DISCUSSION AND REVIEW OF THE CURRENT LITERATURE

We conducted a systemic search for “Mycoplasma hominis,” “lung abscess,” “pleural empyema,” and “lymphoma” in Medline and the Cochrane Library, which returned approximately 80 articles, mostly case reports. Very few and small series with no more than 15 patients have been reported in the literature so far with most patients presenting with identifiable factors that predispose them to infections with *M hominis* such as immunosuppression [2, 6, 17].

*Mycoplasma hominis* is sporadically associated with a broad range of extragenital infections, eg, mediastinitis or endocarditis, bone/joint infections, intrauterine infections, or other abscesses [18, 21, 22]. This may suggest that significant respiratory pathology by “urogenital” mycoplasmas may be more common than generally believed, but it is often simply missed by routine microbiological work-up [18].

Because thoracic surgery patients in general often suffer from underlying chronic respiratory disease, for example, COPD, an increased vulnerability of these patients and especially lung transplant patients can be assumed. The routinely used perioperative bacterial prophylaxis antibiotics, for example, cefepime, are ineffective against *M hominis* [18]. Moreover, most of the current regimens for empirical therapy of posttransplant infectious complications do not cover *M hominis*. Thus, maintaining a high level of suspicion is warranted in critically ill patients presenting with pulmonary infiltrates and negative cultures for respiratory pathogens [11]. Polymerase chain reaction tests may be considered in cardiothoracic patients presenting with pleuritis, surgical site infection, or mediastinitis with negative bacterial cultures or any other suspected bacterial infection with negative Gram stain results [23].

The incidence of hyperammonia in immunocompromised patients is not well known. Therefore, abrupt onset of neurological changes such as lethargy or severe encephalopathy in immunocompromised patients should prompt ammonia-level measurement [24]. Although usually *Ureaplasma* species are the culprits of the ammonium production, infection with *M hominis* has also been associated with hyperammonia in immunocompromised patients [25].

In addition to surgical infectious source control, antibiotic combination therapy of extragenital *M hominis* infections seems to be preferable. However, optimal length of therapy is currently unknown. Although PCR seems useful as a follow-up parameter, it may remain positive for several weeks after appropriate antibiotic treatment [26].

## Conclusions

*Mycoplasma homini*s as a pathogen causing extragenital infections is often underestimated. Therefore, it is very important to contemplate *M homini*s in persistent thoracic infections, especially in immunosuppressed patients or patients with predisposing conditions (eg, surgery or trauma). Diagnosis is difficult and often missed in routine microbiological testing, thus severe extragenital infection with *M hominis* may be underdiagnosed. A heightened suspicion and rigorous precise sampling are pivotal to establish the correct diagnosis and to avoid potentially severe complications for the patient. Given the tendency for chronic infection, long-term antimicrobial treatment should be considered [21], although the optimal length of therapy is currently unknown. Due to a limited number of cases reported so far, further studies are needed to corroborate these findings.
